# Degrading a global COVID-19 contagion: Charting a holistic social work response

**DOI:** 10.1177/0020872821991203

**Published:** 2021-09

**Authors:** Tracy BE Omorogiuwa, Solomon Amadasun

**Affiliations:** University of Benin, Nigeria; University of Benin, Nigeria

**Keywords:** Biopsychosocial, covid-19, person-in-environment, policy advocacy, social work

## Abstract

The eclectic nature of social work in addition to the person-in-environment perspective, as well as its biopsychosocial frame, warrants the utilization of a holistic interventionist lens amid the coronavirus pandemic. This is paramount if we intend to prevent and stymie not just the dreaded contagion in itself, but also its rampaging impact on individuals, families, groups and communities. In this essay, we highlight some empowering framework necessary for action and clarify potential ethical concerns. Given the extensive fallout of the COVID-19 pandemic, illustrative guidelines conducive for holistic professional intervention, during and in the aftermath of the disease, are rolled out.

## Introduction

The eclectic nature of social work in addition to the person-in-environment perspective, as well as its biopsychosocial frame, warrants that holistic intervention be utilizable amid the coronavirus pandemic. This is paramount if we intend to prevent and stymie not just the dreaded contagion in itself, but also its rampaging impact on individuals, families, groups and communities. Using a diagrammatic approach, this essay sets to roll out holistic professional intervention with a view to degrading the after-effects of the coronavirus pandemic (Omorogiuwa, 2020a). Before highlighting these guidelines, it is germane to summarily appraise some empowering framework necessary for our intervention.

Given the biopsychosocial dimension of the COVID-19 pandemic, some theoretical scheme would be germane to aiding our efforts in downgrading the effect of the pandemic. Classic expositions find expression in strengths-based practice (anchored around building hope, resilience, optimism, relationship and resources – some of which are innate and many of which are in the natural environment; Saleebey, 2006), anti-oppressive practice (premised on challenging systemic exclusion, discrimination and marginalization of vulnerable groups; Amadasun and Omorogiuwa, 2020; Baines, 2011; Dominelli, 1996, 2002), rights-based approach (based on challenging happenings of rights violations among vulnerable groups; Amadasun, 2020a; Ife, 2008; Omorogiuwa, 2020a), developmental practice (edged on advocating for investments in social protection programmes [e.g. old age benefits, for older adults] and socioeconomic infrastructures; Amadasun, 2020c; Midgley, 1993, 1995, 2010; Omorogiuwa, 2016, 2020b), and indigenous practice (predicated on according value to indigenous knowledge and practice (e.g. supplementing institutional care with community-based care for at-risk populations).

Are we going to be faced with ethical dilemmas in the course of our work? Most definitely. The ‘holy grail’ of social work ethics: confidentiality and respect for privacy, and self-determination, will be the major ethical issues of concerns. Ethical scholars (Dolgoff et al., 2009; Reamer, 2013; Reamer and Abramson, 1982) have made clear the point that personal or individual interests should succumb to overall public interest, especially when the former has the tendency to wreak havoc on the latter. Although this seems linear and simplistic, it is not always an easy choice in the face of fiduciary or long formed trust between service providers and service users. However, both deontological and teleological thoughts speak of the greatest good for the greatest number, including upholding the sanctity of life of people (Kant, [1797] 1959; Mill, [1861] 1998). By applying this rule, navigating ethical dilemmas may not be so daunting since our primary responsibility is to promote the general well-being of society (National Association of Social Workers [NASW], 2008).

## Roadmap to holistic intervention

**Figure 1.**
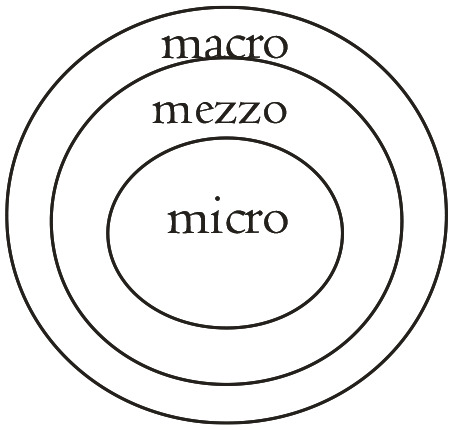
Our potential levels of intervention, transcending the traditional micro and mezzo levels to include the macro level. *Micro*: Individuals. *Mezzo*: Families/households, and groups. *Macro*: Organizations, communities and society as a whole.

**Figure 2.**
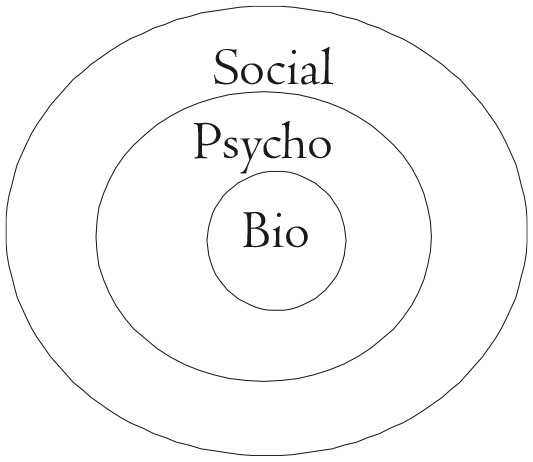
Fallout of the pandemic on the reality realms: biological, psychological and social. *Bio*: Affected, recovery/loss, reintegration/bereavement. *Psycho*: Anxiety, depression, trauma, mental health complications and possibly suicide (Barr et al., 2012; Chan et al., 2014). *Social*: Hunger pandemic (United Nations [UN], 2020 ; Amadasun, 2020d), human rights violations (Agence France-Presse [AFP], 2020; Amadasun, 2020e; Human Rights Watch [HRW], 2020; Khalid, 2020; Kunene, 2020; Sandhu, 2020; Siviwe, 2020), mass layoffs, under- and unemployment (International Monetary Fund [IMF], 2020), loss of self-worth, anxiety, depression, trauma, mental health complications (Omorogiuwa, 2016, 2020), and likely suicide (Barr et al., 2012; Chan et al., 2014).

**Figure 3.**
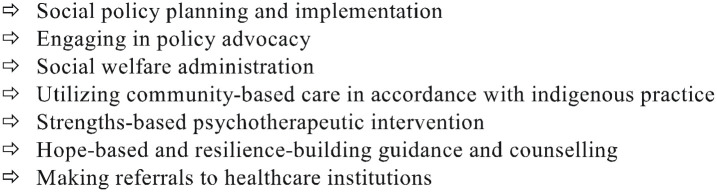
Ladder of holistic professional action.

**Figure 4.**
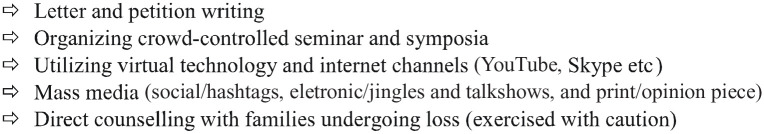
Means of intervention.

The tenacity of COVID-19 has shown that a multifaceted and comprehensive approach (Figures 1 to 4) is needed more than ever to degrade the pandemic. In this regard, in forging alliance and making our voices heard, we can crucially make a meaningful impact in advisorial and advocacy action.

Given that COVID-19 has had a broad impact on all realms, so also should our intervention if we are desirous of preventing the pandemic and degrading its grievous impact on people and society. But in the interim, how can we make meaningful efforts in this regard given the declaration of shutdowns? The following (Figures 3 and 4) will serve as a ladder of holistic intervention, comprising both ad hoc and long-term policy intervention utilizable by social workers and other key stakeholders.

In the context of policy response, Amadasun (2020b) has identified two policy fronts or approaches to policy action: ad hoc and long-term policy action. We equally add a third front: intermediate policy action. Specifically, this should be aimed at evaluative and corrective purposes. Phrased alternatively, it should be oriented towards appraising the effectiveness of ad hoc policy initiative with a view to consolidate on attained objectives while making corrections in the event of policy shortfall.

## Concluding comments

Social workers have continued to display uncommon leadership, bravery and altruism in the face of a deadly virus, defying the odds by putting themselves in harm’s way to serve vulnerable people and defend our collective survival. Yet the scale of our intervention remains unclear. Needless to say, many practitioners are plunged in a state of quagmire as they attempt to navigate ethical dilemmas. Aside from addressing ethical issues and underscoring the imperative of social work intervention, we also rolled out guidelines apt for and conducive to holistic professional action not only amid the global contagion but also in the aftermath of the 2019 coronavirus pandemic.
